# Turning in Arm Presentations

**Published:** 1878-12-20

**Authors:** A. G. Hobbs

**Affiliations:** Ind.


### TURNING IN ARM PRESENTATIONS.

BY A. G. HOBBS, M. D., OF IND.

In September, 1877, I was called in haste to see Mrs. H., a muscular woman, in her second labor. Upon arriving at her bed I found she had been having regular and severe pains for several hours, but they had entirely ceased shortly before my arrival. After sitting about fifteen minutes, she still having no recurrence of pains, I made an examination and found the right hand in the vagina. Had I then followed the precept of our text books I would have proceeded to place my patient under chloroform, at least to the extent of analgesia, and then have introduced my hand into the uterus and brought down the left foot—in short I would have performed podalic version. But I determined to try an experiment, one that would at least not do any injury, if it did not accomplish my purpose. I directed her husband to stand at her feet and raise her limbs and buttox straight up, as in the reduction of hernia. I then had the advantage of gravitation, and by pushing back the arm with the fingers of my right hand in the vagina, and by external manipulations with my left, in less than five minutes the arm was pulled back into the uterus and the head substituted in its place in the superior straight. After a short time natural pains returned, and she was delivered of a healthy boy within an hour after I entered the room.

In March, 1878, I was sent for in haste to see Mrs. A., who had then been in her third labor several hours, and on account of distance it was several hours more before I arrived at her bed. The "granny" remarked that the hand had been born for three hours, which upon examination I found to be true. Pains had ceased for two hours. With but little hopes of success in this case, I resolved to try my former method. Her husband raised her limbs, and the hand was at once drawn back within the vagina, and shortly afterwards within the uterus; then manipulating as before, I succeeded in bringing about a vertix presentation. Labor proceeded, and during the next hour she was safely delivered.

I attribute the success of this method, especially in this case, to the want of tonic contraction of the uterine walls, as the sequel proved, since she had considerable post-partem hemorrhage, which was soon checked by ergot and nux. vom., together with firm external pressure. My object in presenting these two cases is to remind obstetricians that other and less dangerous methods than "*turning*" will sometimes succeed. I do not claim that this method will always prove so successful; we should not expect it to do so when the walls of the uterus are firmly contracted, a condition of the uterus that we by no means always find when the pains have ceased and labor has been at a stand-still for hours. If we do not succeed by this method we have done no harm, and we can then resort to the *dernier*, "*turning*." I do not suppose that I have discovered any thing new in this method, but I am under the impression that it is not generally practiced.

